# Above and below ground carbohydrate allocation differs between ash (*Fraxinus excelsior* L.) and beech (*Fagus sylvatica* L.)

**DOI:** 10.1371/journal.pone.0184247

**Published:** 2017-09-21

**Authors:** Ronny Thoms, Michael Köhler, Arthur Gessler, Gerd Gleixner

**Affiliations:** 1 Max Planck Institute for Biogeochemistry, Jena, Germany; 2 Burckhardt-Institute, Tropical Silviculture and Forest Ecology, Göttingen, Germany; 3 Eidg. Forschungsanstalt WSL, Birmensdorf, Switzerland; Universidade do Minho, PORTUGAL

## Abstract

We investigated soluble carbohydrate transport in trees that differed in their phloem loading strategies in order to better understand the transport of photosynthetic products into the roots and the rhizosphere as this knowledge is needed to better understand the respiratory processes in the rhizosphere. We compared beech, which is suggested to use mainly passive loading of transport sugars along a concentration gradient into the phloem, with ash that uses active loading and polymer trapping of raffinose family oligosaccharides (RFOs). We pulse-labeled 20 four-year old European beech and 20 four-year old ash trees with ^13^CO_2_ and tracked the fate of the label within different plant compartments. We extracted soluble carbohydrates from leaves, bark of stems and branches, and fine roots, measured their amount and isotopic content and calculated their turnover times. In beech one part of the sucrose was rapidly transported into sink tissues without major exchange with storage pools whereas another part of sucrose was strongly exchanged with unlabeled possibly stored sucrose. In contrast the storage and allocation patterns in ash depended on the identity of the transported sugars. RFO were the most important transport sugars that had highest turnover in all shoot compartments. However, the turnover of RFOs in the roots was uncoupled from the shoot. The only significant relation between sugars in the stem base and in the roots of ash was found for the amount (r^2^ = 0.50; p = 0.001) and isotopic content (r^2^ = 0.47; p = 0.01) of sucrose. The negative relation of the amounts suggested an active transport of sucrose into the roots of ash. Sucrose concentration in the root also best explained the concentration of RFOs in the roots suggesting that RFO in the roots of ash may be resynthesized from sucrose. Our results interestingly suggest that in both tree species only sucrose directly entered the fine root system and that in ash RFOs are transported indirectly into the fine roots only. The direct transport of sucrose might be passive in beech but active in ash (sustained active up- and unloading to co-cells), which would correspond to the phloem loading strategies. Our results give first hints that the transport of carbohydrates between shoot and root is not necessarily continuous and involves passive (beech) and active (ash) transport processes, which may be controlled by the phloem unloading.

## Introduction

The transport of photosynthetically fixed CO_2_ as the primary source of organic carbon (C) plays a crucial role in the C cycle of terrestrial ecosystems [1]. From the autotrophic leaves, where photosynthesis takes place, soluble carbohydrates are transported via the phloem to the tissues of C consumption [2]. Approximately 25–63% of the fixed carbon from the gross primary production is transported belowground [3]. The current dominant transport theory is a modified dynamic version of the Muench mass flow model [4]. It assumes a continuous pressure-driven mass flow system in the phloem allowing carbohydrates to be transported over long distances in the plant from source directly to sink tissues. By loading dissolved carbohydrates, especially sucrose, from the source tissue into the sieve tubes of the collection-phloem, and by sugar unloading in the release-phloem of sinks, plants maintain a concentration and thus pressure gradient between source and sink that drives the mass flow [5–7]. The current transport theory suggests an osmoregulatory pressure flow (ORPF), which is determined by the interplay of phloem turgor pressure and osmotic potential of the phloem sap and its surrounding tissues [8–10].

Belowground processes such as soil respiration are closely coupled to photosynthesis in leaves since recent assimilates are known to directly fuel energy consuming reactions in non-photosynthetic tissues (e.g. [11, 12, 13]). The physical mechanisms of this transport through the phloem and their environmental and plant-internal controls are well understood, however, the allocation between plant compartments, the coupling between plant and rhizosphere and thus the control of the ecosystem C balance remain uncertain [14].

During the last few years ^13^C or ^14^C tracer have been applied more frequently to track transport and distribution processes of recently fixed assimilates in plants [14–19]. Calculated mean residence and half-life times of tracers allow quantitative estimates of distribution and storage processes for carbohydrates in plant rhizosphere systems [20]. Tracer studies using temperate species [21–23] demonstrated a rather fast transfer of recently assimilated carbon from leaves to roots and associated soil organisms or fungi [24, 25, 26]. Transport velocities ranged from 0.2 m h^-1^ to 6 m h^-1^ in broadleaved trees. However, these rates are strongly influenced by environmental factors such as soil temperature, soil moisture, tree species and time within the growing season [27]. Even though these labeling studies gave new insights into the effects of environmental and tree internal factors on phloem transport, a mechanistic biochemically based understanding of species-specific transport mechanism is still lacking. This is mainly due to the fact that the recent tracer studies did neither assess the transported sugars nor their label uptake. Studies also rarely combine such compound-specific analyses with the assessment of different plant compartments.

From the assessment of leaf extracts in plants we know, that 50–80% of assimilated carbon are converted to dissolved carbohydrates [28], predominantly to the disaccharide sucrose [5] and to oligosaccharides of the raffinose group (RFO) like raffinose, stachyose and verbascose. Based on carbohydrate concentrations in leaves and non-compound specific transport monitoring, it was hypothesized that different phloem loading strategies exist in trees [29, 30]. Three different phloem loading mechanisms, namely passive loading, polymer trapping (e.g. symplastic loading strategies) and active apoplastic loading to the sieve element companion cell complex have been characterized [31, 32]. The “active” apoplastic loading of sucrose by membrane-bound transporters takes place against a concentration gradient thus leading to an accumulation of carbohydrates in the companion cells and the sieve tubes [5]. Prerequisites for “passive” loading, which is assumed to be important in some trees like *Fagus sylvatica* L., *Quercus rubrum*, *Tilia americana*, are a high abundance of plasmodesmata and likely high sucrose concentrations in the cytosol of mesophyll cells [32]. Symplastic phloem loading is mainly combined with polymer trapping. In this case RFO such as starchyose, verbascose and raffinose are synthesized in the companion cells preventing the RFOs from leaking back into the mesophyll [31, 33, 34]. Previous studies have clearly shown that ash is using either a symplastic (polymer trapping) or mixed symplastic-apoplastic (active upload combined with trapping) loading of the phloem [35]. However, less is known about unloading mechanism for both tree species and it is suggested that they may be comparable to phloem loading mechanisms of the trees [36–38].

Especially the role of RFOs in the phloem unloading and the dependence of RFO concentrations on environmental factors remain unclear. [29] was the first detecting RFO in different plant species especially in ash. Besides being an indicator for polymer trapping, RFOs are found in plants that are sensitive to late frost in spring or early frost in fall [39]. They are produced in response to falling temperature [40, 41]. A temperature dependence of the di-, oligo-and polysaccharide concentrations in conifer shoots and in roots of broad leaf trees has also been shown [40, 42]. Currently, it remains unclear if the RFOs are newly synthesized in the roots as the unloading of RFO from the phloem would involve its breakdown into monomers and sucrose. So far, the prerequisites of RFO resynthesis are present in roots, however, to our knowledge no study exists that investigated the direct transport of RFO into root tissues.

Based on the exsisting knowledge we hypothesize that transport and allocation processes for sucrose in shoot and roots are identical for both plant types and that RFO has to be broken down to sucrose and galactose prior to the release in the root tissue. This would open the possibility for the plants to actively control the RFO efflux into the root and rhizosphere.

## Material and method

### Site description

Our study was conducted in north-eastern part of the Hainich National Park in central Germany close to the village of Weberstedt (51°05´28´´N, 10°31´24´´E) and was permitted by the National Park administration. The National Park is characterized by a mean annual temperature of 7.5°C and a precipitation of 544–662 mm (Data are recorded from four climate stations around the national park; DWD 2008). The study area was located at the altitude of approximately 350 m a.s.l. As a result of a near-natural development with the last timber harvest about 50 years ago, the deciduous forest contains trees of multiple age classes and comprise with high tree species diversity. In the stand studied, the main tree species were European beech (*Fagus sylvatica* L.) and common ash (*Fraxinus excelsior* L.) with a few individuals of other tree species such as *Quercus robur*, *Quercus petrea*, *Prunus avium*, *Ulmus glabra* and *Populus species* [43]. The soil type was classified as Luvisol underlain by limestone. Soil texture was characterized by high silt content over Triassic limestone bedrock [44].

### Labeling and sampling

The experiment was conducted on 25 ash and 25 beech trees. The trees species were similar in height (m; mean± SD) 3.7± 0.4 for beech and 3.1± 0.6 for ash but different in foliage mass (g _DW_) 82.3± 28.6 for beech and 49.9 ± 13.2 for ash. 20 trees of each species were labeled with ^13^CO_2_, while the five remaining individuals served as unlabeled controls. Around each tree we placed a transparent gas tight chamber made of polyethylene foil (thickness of 80 μm). All chambers were 5 m high and 2.5 m in diameter and thus held similar air volumes. They were stabilized with gallows-like wood constructions and closed properly with adhesive tape to avoid gas losses.

Four trees per species were labeled on the 16/08/2011 between 11:30h and 13:30h, eight trees per species on the 17/08/2011 between 9:30h and 12:00h, and another 8 trees per species on 18/08/2011 between 9:30h and 12:00h. The labeling was conducted on sunny days. The photosynthetic photon flux rate on the leaf surface amounted as an average for the different trees and labeling times to 343 ± 123 μmol m^-2^ s^-1^.

We labeled each tree with a pulse of ^13^CO_2,_ which was produced by injecting 60 ml 5M sulfuric acid into a solution of 100 ml distilled water containing 6.85g sodium carbonate (Cambridge Isotope Laboratories, MA, and USA) enriched with ^13^C to 99.0 atom%. The solution was held by a 500 ml polyethylene wide mouth bottle placed in a bowl. Sulfuric acid was added in fivefold excess to ensure complete evolution of ^13^CO_2_ into the atmosphere inside the chamber. The enriched CO_2_ was distributed by a 12 V fan, which was fixed directly above the bottle filled with sodium carbonate. The ^13^CO_2_ exposure was carried out for 5 h and thereafter the chamber was removed.

We harvested four labeled and one control tree per species on day 1, 5, 10, 20 and 60 after labeling separating leaf, stem top, stem base and root material. On a given day the harvesting of all trees was conducted within 12h. 12 leaves were collected from random locations within the tree crown. From 5 cm long branch and stem cuttings the bark was separated from the wood with a scalpel in order to yield mainly phloem tissue. Fine roots were cut from the main rooting system after tearing the tree off the soil. All samples were immediately frozen with liquid nitrogen, kept in boxes filled with dry ice and stored in the laboratory at -80°C.

### Sugar extraction

The plant material was freeze-dried and ground to a fine powder with a ball mill. For each tree, 200 mg of leaf material, 400 mg of branch and stem bark (top stem: material collected directly under the lowest branch (stem top), base stem: material collected approximately 5 cm above the soil surface) and 300 mg of fine root material were placed in bottles and then filled with 20 ml of an ethanol-water mixture (ratio 8:2). Subsequently, each sample was heated to 85°C and then centrifuged to obtain the plant extract. By rotary evaporation, the extract was reduced to about 10 ml to remove the ethanol. This process of consecutive heating, centrifugation and evaporation was repeated three times. In a second step cations and anions where removed by AG MP-50 cation exchange resins columns and by AG MP-1 anion exchange columns, respectively [45].

### Quantification and δ^13^C measurements of carbohydrates

Carbohydrates were separated using a high performance liquid chromatograph (HPLC) (Gilson Inc., Middleton, WI, USA) using a VA/300/7.8 Nucleogel 810 Ca^+^ column. The mobile phase was 100% water with a flow rate of 0.5 ml/min. The column temperature was 80°C. After separation the individual carbohydrates were online oxidized to CO_2_ using ortho-phosphoric acid and sodium peroxidisulfate. The surplus O_2_ was removed with elemental copper and the O_2_ free gas stream was transferred to the mass spectrometer for quantification and stable isotope analysis (Finnigan LC IsoLink Interface, Delta plus XP; Thermo Fisher Scientific, Massachusetts, USA) [44]. The sugars were identified by their retention time using external sugar standards. The concentrations of individual sugars in mg C were calculated from the mass spectrometric CO_2_ signal using external standard calibration. The amount and isotopic compositions of the RFOs were calculated from the weighted isotopic mean of raffinose, verbascose and stachyose that were not fully separated on the column.

### Isotopic calculations

Isotopic values are expressed in the δ notation (‰), relative to the Vienna Pee Dee Belemnite standard. To estimate the amount of ^13^C added by pulse labelling, the isotopic abundance of the labelled samples was converted to atom% ^13^C and the atom% excess was calculated by subtracting the atom% ^13^C values of the unlabelled controls.

In addition, the total ^13^C (mg ^13^C g^-1^ DW) was calculated as atom% excess multiplied by the amount of carbohydrates in each sample [46]. Differences in total ^13^C (mg ^13^C g^-1^ DW) between different times after labeling were assessed with one way ANOVA of repeated measures followed by Post-Hoc Tukey tests. The data were log-transformed to ensure normal distribution of residuals.

### Calculation of ^13^C residence time

Mean residence and half-life times were determined by an exponential decay function [47] fitted to the data using the nls package of the statistic program R (version 2.11.1; R Development Core Team, 2011):

$$N\left(t\right)=N*e^{-\lambda {\;}_{Ι}\;\tau }$$ (1)

where *τ* is the time at that label peaked, N describes the fraction of ^l3^C at peak time, *λ*_I_ is the constant rate of tracer loss and N(t) is the fraction of ^13^C at time t. The mean residence time (i.e. the average time that C atoms remain in a defined compartment) can be calculated as

$$t=1/\lambda_{Ι}$$ (2)

The half-life time (50% of C atoms are left in a defined reservoir) is given by [18]:

$$t_{1/2}=\text{ln}\;\left(2\right)/\lambda_{Ι}$$ (3)

although, not being a precise measure of goodness of fit of nonlinear models, R^2^_adj_ is given to allow comparisons with estimates of other studies.

## Results

### Carbohydrate ^13^C dynamics in beech and ash

**Fig 1:**
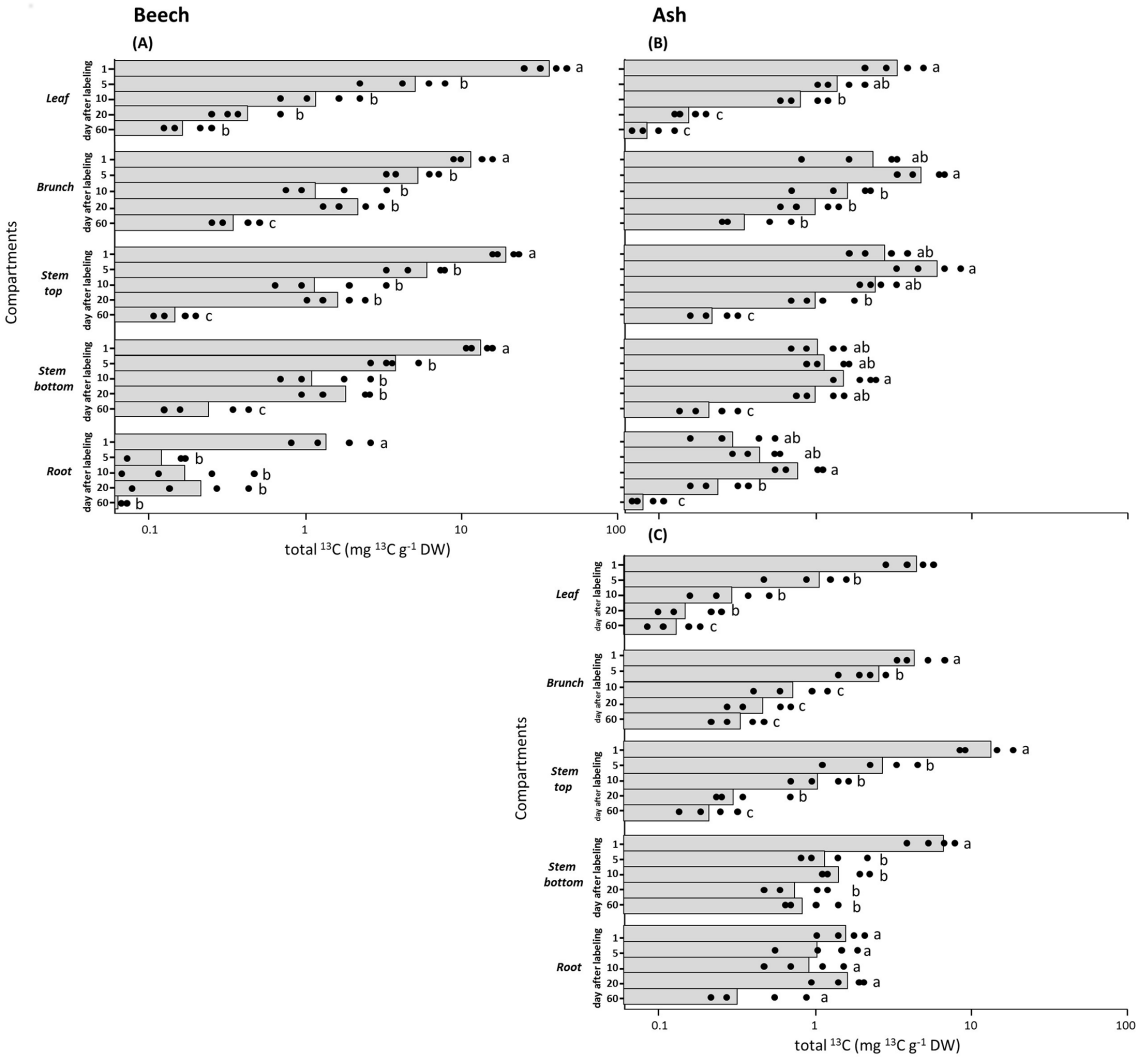
Enrichment of total ^13^C in the main carbohydrates sucrose and raffinose group (RFO) in different plant compartments of beech (*Fagus sylvatica*) and ash (*Fraxinus excelsior*) from 1 day (17.08.2011) to 60 days after labeling. Black dots refer to different replicates of each time point for sucrose in beech **(A)** sucrose in ash **(B)** and RFO in ash **(C).** Different letters indicate significant differences (p ≤ 0.05) among days after labeling for a given carbohydrate (repeated measure ANOVA followed by post-hoc Tukey HSD test). (see also supporting information S1 Table).

In beech, sucrose was the main sugar that was ^13^C labelled and transported from leaves to other compartments (96% of the ^13^C label). We also found minor proportions of ^13^C in glucose (3%) and fructose (1%). Already one day after labelling, the highest ^13^C label was found for sucrose in all studied compartments of beech. Thus, the label peak must have occurred even before our first sampling. The enrichment was 28 fold higher in leaves (37 mg ^13^C g^-1^ DW total ^13^C) compared to roots (1.3 mg ^13^C g^-1^ DW total ^13^C, Fig 1). The enrichment decreased strongly between the first and second time point in the leaves, less pronounced in the branches, and did not change significantly between day 5 and day 60 after labeling in the roots (Fig 1). The temporal dynamic of the ^13^C label was very similar for all compartments in beech (Fig 1). The mean residence (MRT) of sucrose in leaves and roots was around 2 d and slightly higher in the stem (3.4 d) and the branch (5 d) (Table 1).

**Table 1:** Mean residence and half-life time of ^13^C in main carbohydrates of beech and ash trees in different compartments leaf, branch, stem and root. The data was fitted to a one-pool model given a coefficient of determination R^2^adj. and the p-value. The mean residence and half-life time were estimated based on an exponential decay function. (see also supporting information S1 Fig).

compartment	*sucrose ash*				*raffinose group ash*				*sucrose beech*			
	MRT (d)	HLT (d)	R^2^_adj._	p	MRT (d)	HLT (d)	R^2^_adj._	p	MRT (d)	HLT (d)	R^2^_adj._	p
*leaf*	***4*.*8***	***3*.*3***	*0*.*98*	*0*.*001*	***2*.*8***	***1*.*9***	*0*.*90*	*0*.*001*	***2*.*0***	***1*.*4***	*0*.*91*	*0*.*001*
*branch*	***5*.*1***	***3*.*5***	*0*.*93*	*0*.*023*	***6*.*3***	***4*.*3***	*0*.*96*	*0*.*002*	***5*.*0***	***3*.*4***	*0*.*93*	*0*.*005*
*stem top*	***5*.*8***	***4*.*1***	*0*.*98*	*0*.*006*	***2*.*6***	***1*.*8***	*0*.*99*	*0*.*001*	***3*.*4***	***2*.*3***	*0*.*90*	*0*.*001*
*stem base*	***14*.*9***	***10*.*4***	*0*,*98*	*0*,*002*	***2*.*8***	***1*.*9***	*0*.*85*	*0*.*010*	***3*.*4***	***2*.*3***	*0*.*90*	*0*.*002*
*root*	***7*.*9***	***5*.*5***	*0*.*91*	*0*.*007*	***25*.*6***	***17*.*7***	*0*.*82*	*0*.*060*	***1*.*8***	***1*.*2***	*0*.*91*	*0*.*007*

In the source tissue of ash we observed that approx. 88% of ^13^C label was distributed to RFOs and sucrose, while in monosaccharide we found approx. 12%. The consecutive decay of ^13^C enrichment in RFOs in the ash shoot was similar to sucrose in beech with highest enrichments at day one and continuous depletion afterwards (Fig 1). The ^13^C enrichment of RFOs in the root did, in contrast to the shoot, not significantly differ between day 1 to day 60 (Fig 1E). Sucrose had only in ash leaves the consecutive decay of the ^13^C enrichment like it was observed for RFOs (Fig 1A). In all other compartments the ^13^C enrichment was highest between day 5 and day 10 (Fig 1).

The half-life (HLT) and mean-residence times (MRT) of ash RFOs were comparable to that of beech sucrose, whilst the HLT and MRT of sucrose in ash leaves were much higher than that of beech leaves (Table 1). In branches, MRT and HLT were comparable between RFO and sucrose in ash and broadly in the same range as sucrose in beech. In the stem, the MRT and HLT of RFOs in ash were comparable to sucrose in beech, whilst the turnover of ash sucrose was between 1.1- and 4.4-fold slower. In ash roots MRT and HLT of both, RFO and sucrose were clearly higher than the values for sucrose in beech with the highest value for RFO amounting to 25.6 days. We thus encountered similar time-dependent labeling patterns for sucrose and RFO at the location of photosynthesis in ash (Fig 1A) ii) higher and HLT for sucrose in the stem where intermediate storage might occur (Table 1) and iii) contrastingly higher MRT and HLT of RFOs than sucrose in the roots, which are the final sink tissues (Table 1).

### Carbohydrate concentrations in ash and beech

**Fig 2:**
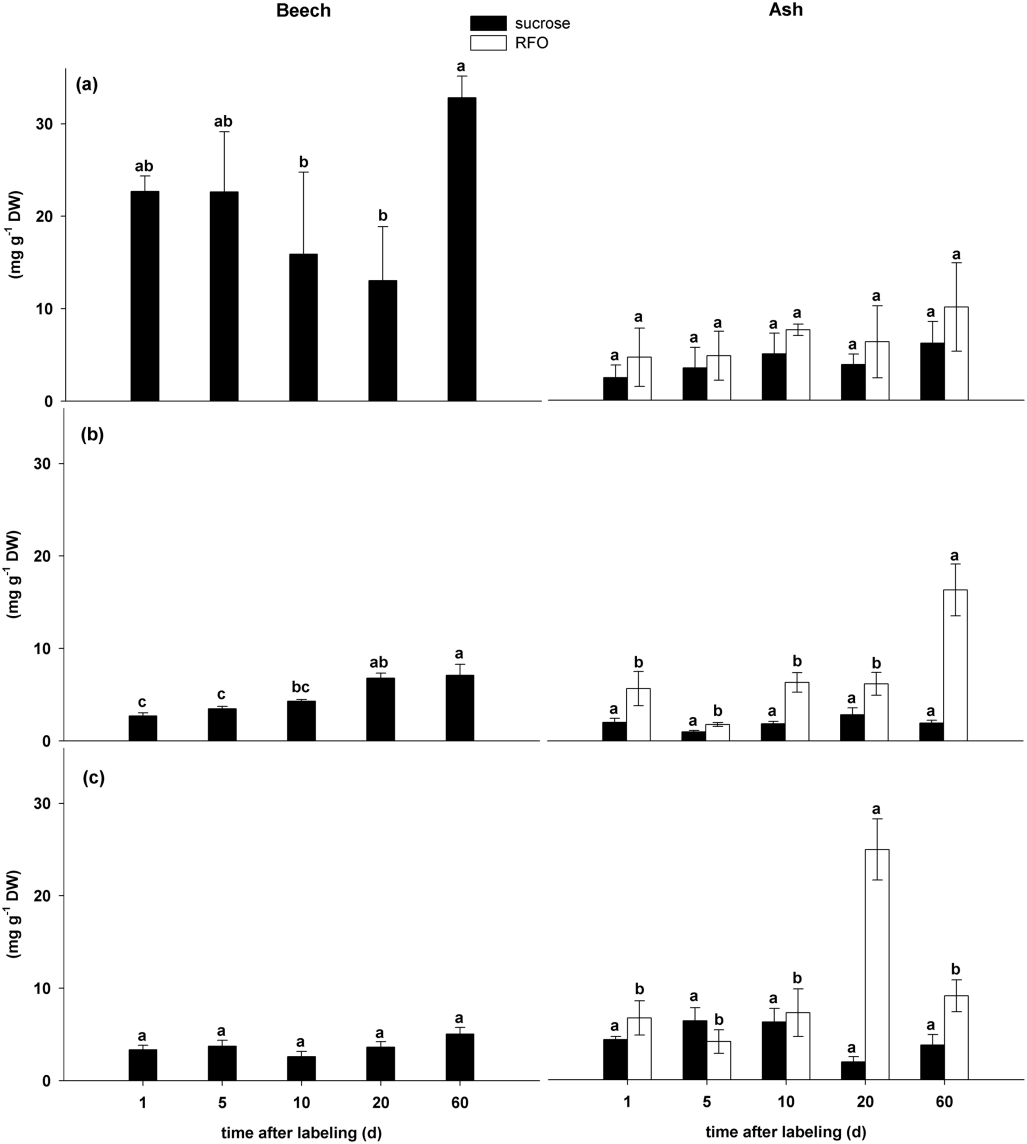
Concentration of the carbohydrates RFO and sucrose in beech and ash trees in different compartments (a) leaf, (b) stem base and (c) root from 1 day to 60 days after labeling (mean values ± SD, n = 4). Different letters indicate significant differences (p ≤ 0.05) among days after labeling for a given carbohydrate (repeated measure ANOVA followed by post-hoc Tukey HSD test).(see also supporting information S2 Table).

Sucrose concentration (mg g^-1^ DW) was much higher in beech leaves than in all other beech compartments as it is likely for passive phloem loading. The concentrations varied over time and were highest on day 60 (17.10.2011) (Fig 2a). In stems and roots of beech the sucrose concentration varied less and increased in stem base but not for the roots with time (Fig 2b and 2c).

In ash, the concentrations of RFO and sucrose in source and sink tissues showed different temporal patterns (Fig 2a and 2c): While the concentration of sucrose was very similar in all compartments and over time, the concentration of RFOs expectedly increased to the end of the growing season in the stem and in the root [48–50]. The RFO concentration changed rapidly in roots and stem with maximum values of 10 mg g^-1^ DW in stems at day 60 (17.10.2011) and 15 mg g^-1^ DW in roots at day 20 (06.09.2011). Simultaneously, we observed a insignificant decrease of sucrose concentration from 4 mg g^-1^ at day 10 (26.08.2011) to 1 mg g^-1^ at day 20 (06.09.2011).

### Relation of carbohydrates in shoot and root of ash and beech

**Fig 3:**
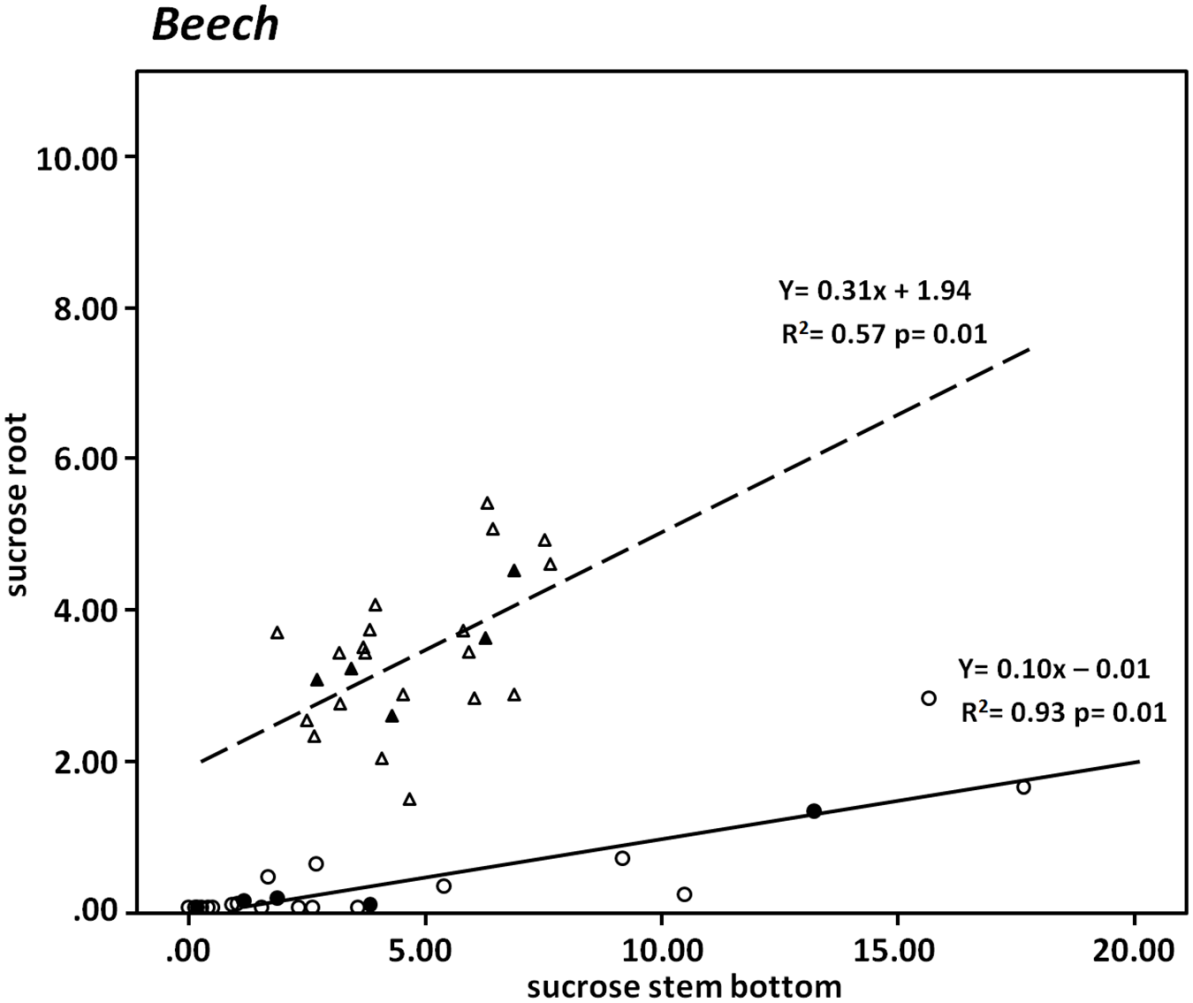
Relationship between sucrose in compartments stem base and root in beech. The solid line shows the total ^13^C enrichment [mg ^13^C g^-1^DW] and the dashed line shows the concentrations [mg g^-1^DW]. The filled symbols represent the mean values based on the independent replicates (open symbols).

The concentration of sucrose in the stem of beech was always higher than the concentration of sucrose in the roots and both concentrations were significantly correlated to each other (Fig 3). The isotopic enrichment of sucrose in the stem was about ten times higher than in the roots and both were significantly correlated to each other (Fig 3).

**Fig 4:**
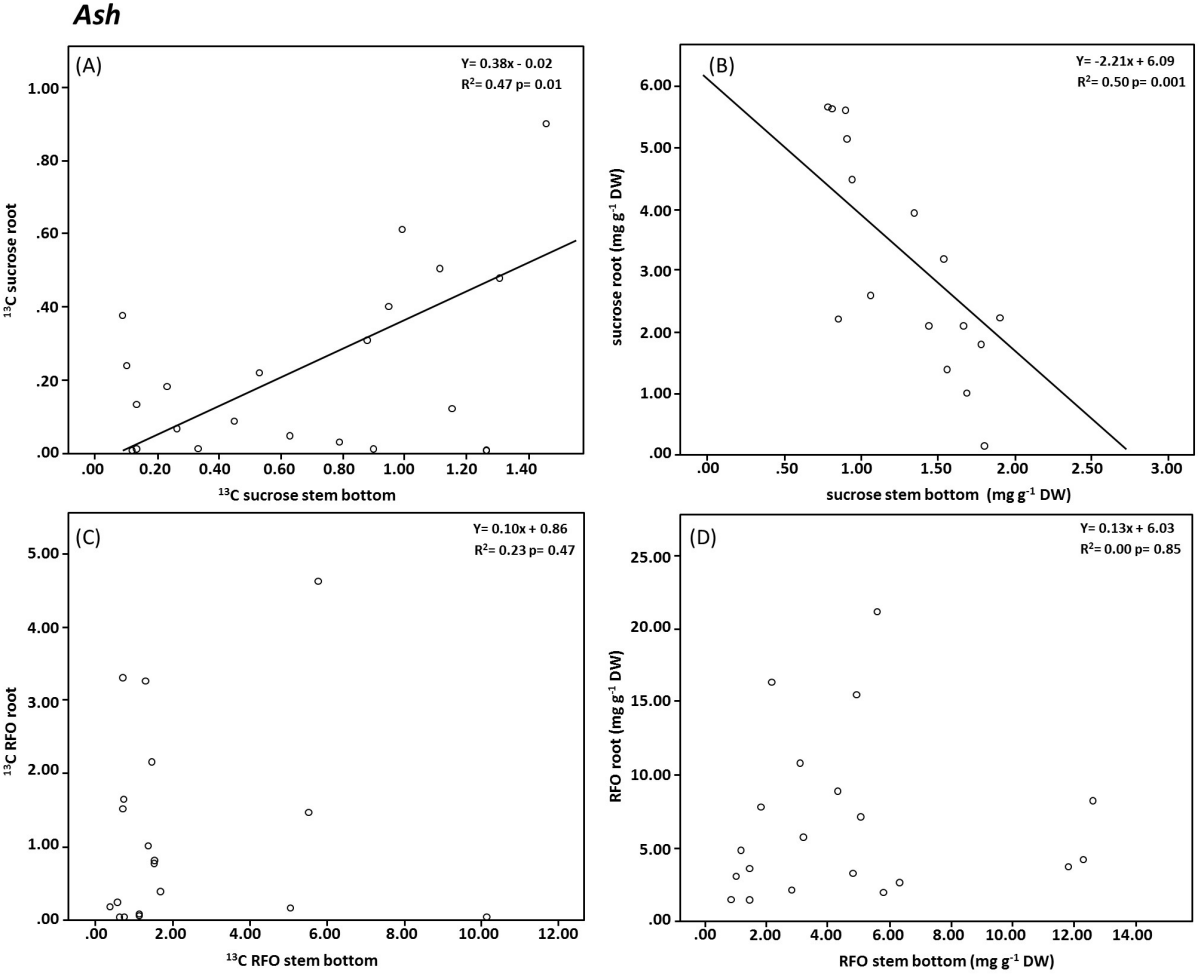
Relationship between above ground (stem base) and belowground compartments (root) for total ^13^C enrichment [mg ^13^C g^-1^DW] in sucrose (A) and in RFOs (C) and concentration [mg g^-1^DW] (B) and (D). The filled symbols represent the mean values based on the independent replicates (open symbols).

**Fig 5:**
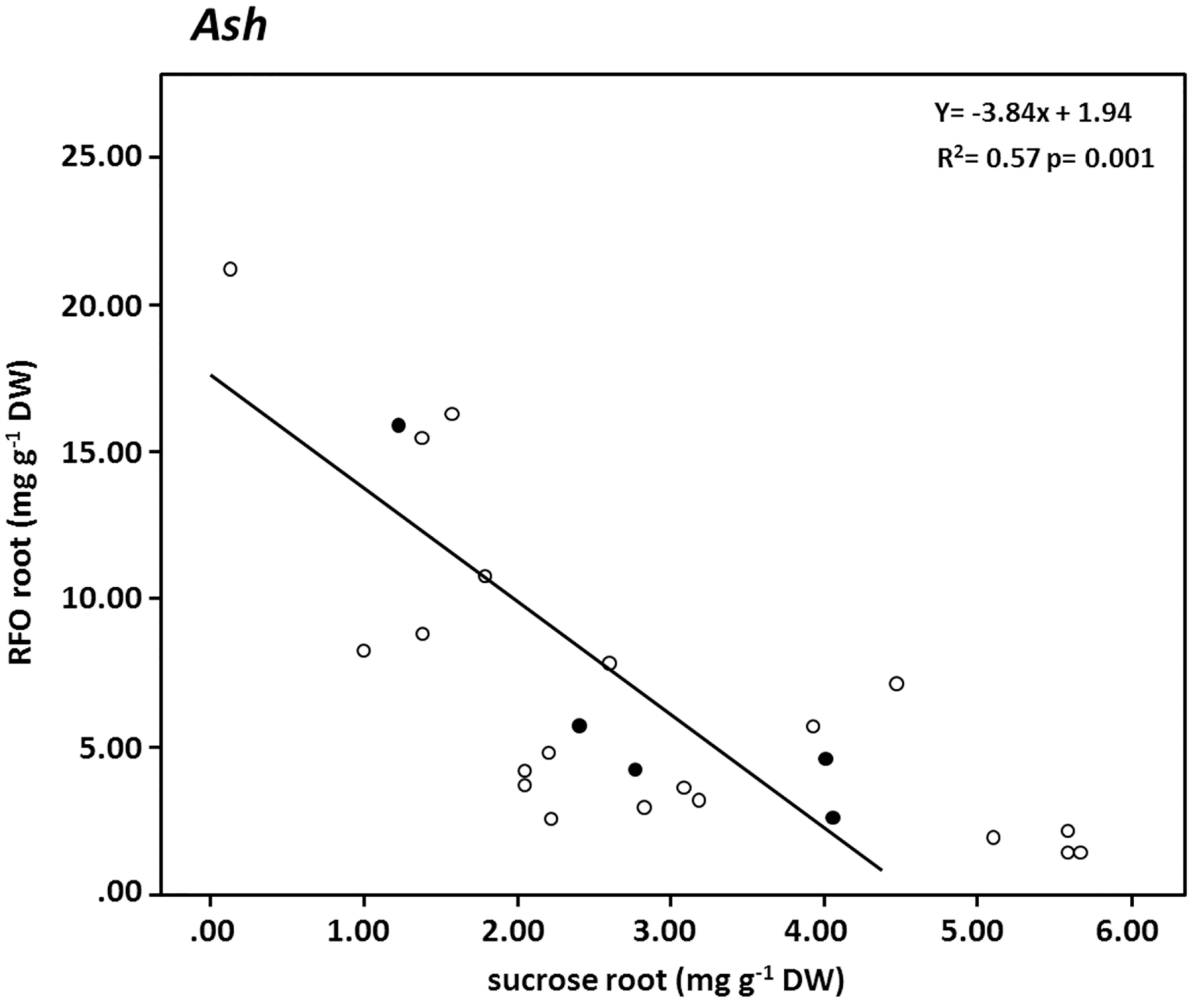
Relationship between RFO and sucrose concentrations in compartment root in ash. The filled symbols represent the mean value based on independent replicates (open symbols).

Significant correlations were also found between stem and root sucrose in ash (Fig 4). The isotopic enrichment was, like in beech, higher in the stem than in the root and both were positively correlated. The overall concentration of sucrose, in contrast, was mostly higher in the root than in the stem and both were negatively correlated (Fig 4B). Unexpectedly no correlation was found for RFO concentration and their isotopic enrichment between both compartments (Fig 4C and 4D). Particularly interesting was however the relationship between RFO and sucrose within the root tissue of ash trees. Here, we found a negative significant correlation between both carbohydrates (Fig 5).

## Discussion

Many studies of carbohydrate allocation found rapid transport rates of freshly assimilated carbon in beech and ash (0.2–1.3 m h^-1^) [18, 51]. In these studies transport velocity is calculated from the time difference between the uptake of labeled ^13^CO_2_ and the first appearance of ^13^C in soil respiration. The calculated transport rates do not provide, however, information about storage, transport processes, the physiology involved as well as unloading mechanisms in different tree species. As a consequence we assessed the dynamics of carbohydrate transport compound-specific and compartment-wise in order to get a more detailed understanding of the mechanisms driving carbon transport in different species.

### Dynamics of ^13^C label in plant carbohydrate pools in beech

In beech we observed the highest ^13^C enrichment of sucrose already on the first day after labeling in all compartments (leaf, branch, stem top, stem base and root) (Fig 1). This suggests a high transport rate from the source to the sink like typically mentioned in the literature for sucrose in beech [16] This is also supported by similar and short mean residence times of sucrose in beech leaves and roots (Table 1). Still, the lacking temporal replicates do not allow for a more precise estimate of transport velocity.

However, the turnover time of sucrose was much higher in the other beech compartments: The mean residence time (5.0 d) and half-life time (3.4 d) in the branches and in the stem (3.4 d and 2.3 d, respectively) were 2–3 times higher compared to leaves and roots (Table 1). This might be caused by labeled sucrose being temporarily stored within the stem and exchanged with unlabeled pools. Besides that, concentration of sucrose in roots can be lower due to its utilization: the concentration of non-structural carbohydrates is generally negatively correlated to growth intensity [50].

The significant positive correlation of sucrose concentrations in connection with the ^13^C enrichment distribution between both compartments (Fig 3) suggests a passive, no energy dependent, transfer mechanism that followed the concentration. As a consequence our results support that in beech two different transport components exist. One which is associated with a fast transport of carbohydrates from the source to the roots and the rhizosphere [52] and the other one, which is in exchange with intermediate storage pools. This suggests that the carbohydrate transport on the one hand under photosynthetic (source) control and on the other hand under the control of the internal storage pools [53]. The two transport components would enable beech to maintain high exudation rates of carbohydrates in order to support their beneficial ectomycorrhiza [52], [54].

### Dynamics of ^13^C label in plant carbohydrate pools in ash

In ash we have indications for different transport mechanisms that may be linked to the different transport sugars of ash namely sucrose and RFO [29, 55]. The reason might be the combined use of symplastic (polymer trapping) and apoplastic (active sucrose) phloem loading mechanisms [35]. This assumption is supported by the dynamics of both sugars. RFO hold higher ^13^C enrichment and have faster turnover rates than sucrose, and both sugars have comparable temporal ^13^C enrichment in the leaves and the branch (Fig 1A and 1B). This likely indicates that in source tissues polymer trapping might be more important for phloem loading than the apoplastic loading.

However, both sugars change differently and have a different temporal pattern in sink tissues like stem and root (Fig 1C, 1D and 1E). The lateral exchange (Table 1) and peak enrichment (Fig 1) is much slower and later for sucrose, respectively. This finding supports ongoing transport of sucrose in and out of the phloem along the transport path [48], which would delay and dampen the ^13^C signal of sucrose. But it is clear from our data that RFO in the stem are not part of this sugar exchange as they still show the same dynamic like in the source tissues (Table 1, Fig 1).

Most striking is, however, the kinetic disconnection of RFO in the roots of ash. On the one hand RFO have a comparable ^13^C signal like sucrose in beech (Fig 1). This indicates a fast transport of RFO to the roots and might be an indication for RFO not being passed to intermediate storage pools.

### Connection between above and belowground carbohydrate pools of ash

If we analyze the sugar pattern between the stem base and the root in more detail (Fig 4), we observe no correlation between RFO in the stem and in the root; neither for sugar concentrations (Fig 4D) nor for their isotopic enrichment (Fig 4C). This suggests that both pools are not directly related to each other and consequently that RFO are not directly transferred into the root tissue. However, sucrose in the stem is highly correlated to the sucrose concentration in the roots (Fig 4B). This negative correlation, the correlation in the label transfer (Fig 4A) and the generally higher sucrose concentrations in the roots compared to the stem base (Figs 2 and 4B) suggest an active (against the concentration gradient) transport process for sucrose into the root tissue. This would be in line with our hypothesis that active phloem loading and unloading strategies exists for sucrose in ash and it would support the findings of [56], who concluded that only an active symplastic unloading mechanism with an apoplastic step (i.e. a carrier especially for sucrose) is used for the transport of carbohydrates from the phloem to sink cells. Furthermore such an active transfer of sucrose to the root might enable the regulation of the carbon flow to the roots and their symbiotic arbuscular mycorrhizal fungi.

However, if we assume that the RFOs remain in the stem and only sucrose is actively unloaded into ash roots, then the question arises: Why do we detect RFOs (and ^13^C incorporation into RFOs) in fine root tissue? Most likely RFOs are resynthesized from sucrose and galactose molecules in the roots again. This hypothesis is supported on the one hand by the presence of specific transport proteins that transport galactose molecules through the membranes of sink tissue [49] and on the other hand by the negative correlation between sucrose and RFOs concentration in roots (Fig 5). Such a root internal process would enable roots to synthesize RFO as anti freezing agents independent from the carbohydrate transport [41, 50].

## Conclusion

Our new approach that combined in-situ ^13^C pulse label experiments of whole trees with compound-specific ^13^C measurements of carbohydrates from different compartments of trees that differed in their transport strategies gave new and unexpected insight in the carbon allocation of trees. In both tree species we found differences in transport and storage processes between aboveground and belowground parts.

In beech, two different transport components likely transport the main carbohydrate sucrose. One component directly and fast transports sucrose into root tissues without further exchange with other sink tissues. The other component involves strong interactions with sink / storage tissues. In consequence beech trees may support a high transport rate of sucrose directly to the root tissues and the rhizosphere using both newly synthetized and stored sugars. This mechanism may help beech i) to promote growth at stress situations, like drought induced root growth of beech and ii) to create via high root exudation rates favorable conditions for their own growth.

In ash, the main transport sugars sucrose and RFO are treated differentially. Sucrose in the phloem seems constantly exchanged with other (parenchymatic) cells during transport, while RFOs are preferentially kept in the phloem but not directly transferred into fine root tissues. As a consequence, the export of sugars to the fine root tissue seems to be regulated via active sucrose unloading and it remains to be clarified if this regulation is linked to the presence and carbon demand of endomycorrhiza fungi.

In the future, more research on metabolic fluxes is needed to better understand the link between phloem transport and how plants control the rhizospheric activity.

## Supporting information

S1 Table Enrichment of total ^13^C in the main carbohydrates sucrose and raffinose group (RFO) in different plant compartments of beech (*Fagus sylvatica*) and ash (*Fraxinus excelsior*). (PDF)

S2 Table Concentration of the carbohydrates RFO and sucrose in beech and ash trees in different compartments. (PDF)

S1 Fig Calculation of mean residence and half life times. (TIFF)
