# Supplementary material for: Above and below ground carbohydrate allocation differs between ash (Fraxinus excelsior L.) and beech (Fagus sylvatica L.)
Source: PLoS One. 2017 Sep 21;12(9):e0184247. doi: 10.1371/journal.pone.0184247 (PMC5608211; doi:10.1371/journal.pone.0184247)
Supplement: S1 Table — (PDF) [file pone.0184247.s001.pdf]

time	beech su. leaf	beech suc. branch	beech suc. stem top	beech suc. stem bottom	beech suc. roots	ash suc. leaf	ash suc. branch	ash suc. stem top
1	34.41	8.97	15.49	10.43	0.23	0.96	0.20	0.88
1	23.77	7.78	14.91	15.60	2.85	1.45	0.58	0.85
1	48.63	15.28	25.13	17.63	1.66	2.56	2.80	2.29
1	41.32	13.22	22.32	9.17	0.73	4.69	2.96	3.95
5	6.37	2.96	6.61	3.76	0.03	0.74	5.27	0.68
5	8.50	8.32	6.91	5.36	0.36	0.61	2.70	6.28
5	3.29	3.46	3.80	3.58	0.03	0.94	3.29	9.35
5	2.10	5.97	7.02	2.65	0.06	1.39	2.77	1.92
10	1.31	0.25	0.25	0.34	0.07	0.41	1.28	1.39
10	0.47	1.25	0.36	0.96	0.12	1.26	0.27	1.85
10	0.79	2.62	2.66	1.66	0.49	0.15	1.70	2.24
10	2.13	0.50	1.31	1.46	0.01	0.21	1.15	1.46
20	0.88	1.72	1.65	1.52	0.01	0.10	0.24	0.46
20	0.23	3.83	1.71	2.72	0.65	0.05	0.89	0.25
20	0.27	1.26	1.51	0.86	0.11	0.10	1.09	1.61
20	0.35	1.72	1.64	2.29	0.11	0.08	0.38	0.28
60	0.06	0.42	0.04	0.05	0.04	0.04	0.13	0.09
60	0.09	0.49	0.07	0.34	0.07	0.05	0.32	0.21
60	0.13	0.26	0.15	0.15	0.05	0.03	0.13	0.14
60	0.37	0.19	0.32	0.43	0.09	0.05	0.25	0.06

time	ash suc. stem bottom	ash suc. root	ash RFO leaf	ash RFO branch	ash RFO stem top	ash RFO stem bottom	ash RFO root
1	0.36	0.18	2.27	0.53	5.37	5.54	1.46
1	0.23	0.03	3.81	3.56	6.41	5.06	0.16
1	0.79	0.48	5.76	4.35	14.18	5.80	4.61
1	1.31	0.01	6.16	8.84	27.87	10.13	0.02
5	0.33	0.40	0.56	6.41	0.41	1.50	0.77
5	0.95	0.05	1.17	1.80	2.58	0.66	0.01
5	0.63	0.50	0.56	1.32	7.32	1.33	3.25
5	1.11	0.12	1.95	0.70	0.65	1.12	0.05
10	1.15	0.90	0.32	0.54	1.01	1.48	2.15
10	1.46	0.61	0.44	0.14	0.86	1.38	1.02
10	0.99	0.09	0.22	0.49	0.89	1.13	0.08
10	0.45	0.31	0.18	1.70	1.45	1.68	0.38
20	0.88	0.22	0.09	0.13	0.30	0.72	1.52
20	0.53	0.01	0.19	0.65	0.12	0.76	0.02
20	0.90	0.07	0.25	0.66	0.49	0.73	1.65
20	0.26	0.24	0.07	0.41	0.28	0.74	3.31
60	0.10	0.01	0.11	0.18	0.13	0.40	0.18
60	0.13	0.00	0.16	0.66	0.21	0.76	0.01
60	0.11	0.13	0.06	0.24	0.38	1.54	0.80
60	0.13	0.01	7.90	0.25	0.13	0.59	0.24
