# Supplementary material for: Above and below ground carbohydrate allocation differs between ash (Fraxinus excelsior L.) and beech (Fagus sylvatica L.)
Source: PLoS One. 2017 Sep 21;12(9):e0184247. doi: 10.1371/journal.pone.0184247 (PMC5608211; doi:10.1371/journal.pone.0184247)
Supplement: S2 Table — (PDF) [file pone.0184247.s002.pdf]

time	beech su. leaf	beech suc. stem bottom	beech suc. roots	ash su. leaf	ash suc. stem bottom
1	25.18	1.90	3.66	1.73	0.62
1	21.94	3.21	2.76	1.75	1.55
1	21.67	3.19	3.43	1.77	1.07
1	21.98	2.54	2.52	4.48	1.90
5	28.55	3.79	3.70	0.92	0.41
5	22.81	3.74	3.39	2.39	0.79
5	25.68	3.69	3.50	4.76	0.47
5	13.54	2.68	2.34	5.85	0.82
10	9.75	4.69	1.48	4.95	1.57
10	11.85	4.53	2.86	8.21	1.34
10	12.88	3.93	4.03	3.45	0.90
10	29.07	4.08	2.03	3.41	0.91
20	17.10	6.83	2.87	4.62	1.79
20	9.24	6.31	5.38	4.44	1.81
20	18.90	6.05	2.82	4.21	2.99
20	6.93	5.93	3.45	2.19	0.67
60	32.76	5.81	3.70	6.81	0.95
60	30.88	6.42	5.04	7.96	1.67
60	36.11	7.51	4.90	2.69	0.90
60	31.54	7.66	4.58	7.16	1.44

time	ash suc. roots	ash RFO leaf	ash RFO stem bottom	ash RFO roots
1	3.09	2.84	1.45	3.56
1	3.18	3.60	4.83	3.22
1	2.59	2.80	1.82	7.76
1	2.23	9.33	6.35	2.54
5	2.83	1.29	1.02	2.95
5	5.66	7.31	0.87	1.41
5	2.21	4.34	1.18	4.83
5	5.60	6.26	1.48	1.38
10	1.38	7.84	4.32	8.83
10	3.92	7.93	3.22	5.69
10	5.58	8.00	2.83	2.10
10	5.10	6.70	5.82	1.92
20	1.80	5.59	3.11	10.80
20	0.15	9.26	5.57	21.12
20	1.37	9.31	4.94	15.46
20	1.59	1.09	2.19	16.28
60	4.47	10.20	5.09	7.13
60	2.06	12.01	11.79	3.69
60	1.01	3.46	12.59	8.19
60	2.06	14.65	12.27	4.18
