# Supplementary figures and images for: Above and below ground carbohydrate allocation differs between ash (Fraxinus excelsior L.) and beech (Fagus sylvatica L.)
Source: PLoS One. 2017 Sep 21;12(9):e0184247. doi: 10.1371/journal.pone.0184247 (PMC5608211; doi:10.1371/journal.pone.0184247)

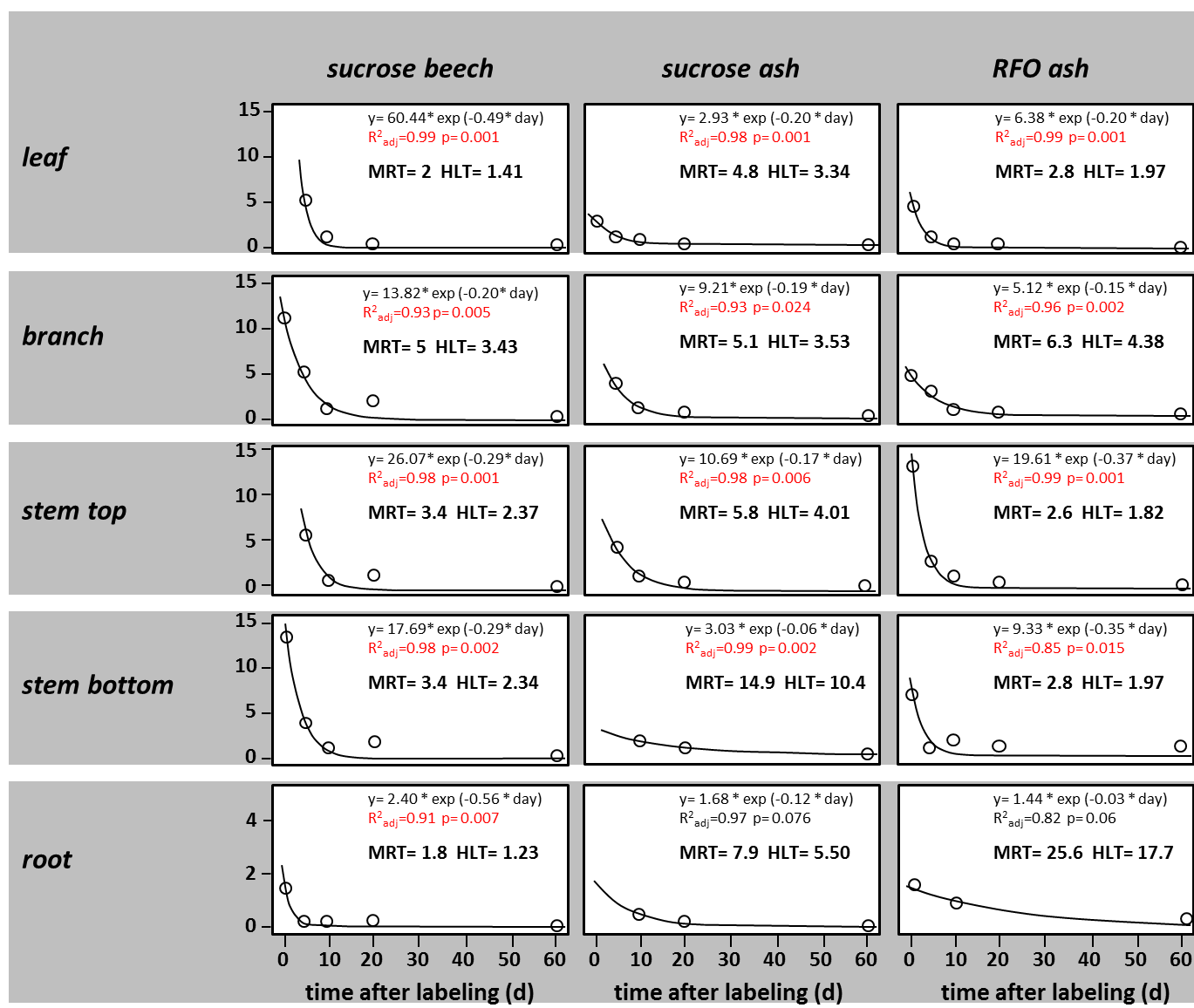

Supplement: S1 Fig — (TIFF) [file pone.0184247.s003.tiff]
